# Modified Test Kit for Detecting Polar Compounds and Evaluating Their Distribution in Reused Frying Oil

**DOI:** 10.3390/foods14091572

**Published:** 2025-04-29

**Authors:** Rapeepan Yongyod, Anusak Kerdsin

**Affiliations:** Faculty of Public Health, Kasetsart University Chalermphrakiat Sakon Nakhon Campus, Sakon Nakhon 47000, Thailand; anusak.ke@ku.th

**Keywords:** polar compounds, reused frying oil, modified test kit, oil type

## Abstract

Polar compounds in repeatedly used frying oil pose significant health risks to consumers. This study aimed to develop an improved test kit for detecting polar compounds in used frying oils and to compare the distribution of polar compounds across different types of cooking oils. The modified test kit was evaluated using six types of oils, which were heated and tested against a standard method with 100 samples. The modified test kit demonstrated an accuracy of 92.00%, sensitivity of 88.09%, specificity of 94.82%, positive predictive value of 92.50%, and negative predictive value of 91.66%. The polar compound distribution was analyzed in six types of oils: palm oil, coconut oil, rice bran oil, sunflower oil, canola oil, and soybean oil. Coconut oil was found to be the least suitable for frying due to the rapid formation of polar compounds. In contrast, rice bran oil, sunflower oil, canola oil, and palm oil were more suitable for frying, with polar compound contamination occurring only after more than 80 h of use. These findings can assist food service operators in extending oil usage while ensuring consumer safety.

## 1. Introduction

Deep-fat frying is a widely used cooking method that significantly alters the chemical and physical properties of oils and foods. It generates both desirable and undesirable flavor compounds, affects flavor stability, color, and texture, and may degrade the nutritional quality of fried foods. During frying, oils undergo common chemical reactions, such as hydrolysis, oxidation, and polymerization, leading to the formation of a wide range of volatile and non-volatile compounds [1]. Various factors accelerate these reactions, including exposure to air, high temperatures, frequent reuse, extended frying times, and high saturated-fatty-acid content in the oil [2,3]. These reactions cause triglycerides to break down, forming various compounds, including volatile substances such as hydrocarbons, aldehydes, ketones, alcohols, and acids. Non-volatile compounds are also formed, which include non-polar compounds (without charge or polarity) such as oligomers and polymers of triglycerides, and polar compounds (with charge or polarity) such as free fatty acids, oxidized fatty acids, monoglycerides, diglycerides, and oxidized monomers, dimers, or polymers of triglycerides [4,5].

The formation of polar compounds in oil leads to its degradation, with higher polar compound levels indicating more advanced degradation [6]. As polar compound concentrations increase, the oil becomes viscous, prone to foaming, develops a lower smoke point, darkens in color, and produces undesirable odors. Additionally, toxic substances harmful to health are formed [7]. These toxins can contribute to the development of cancer and may lead to cardiovascular diseases, heart disease, and hypertension [8]. Not only are consumers at risk, but food service workers are also exposed to hazards, as vapors from degraded oils can contain lung carcinogens [8].

In Thailand, safety standards for oil use are defined by the Ministry of Public Health, (Announcement No. 283 B.E. 2547, 2004) [9], which states that oils used for frying or food preparation for sale should not contain more than 25% polar compounds by weight [10]. Polar compounds can be measured using column chromatography, which involves dissolving an oil sample (approximately 2.5 g) in a mixture of petroleum ether and diethyl ether (87:13 ratio) to a final volume of 50 mL. A 20 mL aliquot of this solution is passed through a silica gel column, where polar compounds are adsorbed. The non-polar compounds are eluted with 150 mL of the solvent mixture, collected, evaporated to dryness, and weighed. The quantity of polar compounds is then calculated as a percentage of the original sample weight.

However, this laboratory analysis requires specialized equipment and expertise, making it impractical for routine use. The process is time-consuming, taking 1–2 days to complete, and the equipment is costly. Other polar compound test kits are expensive at about USD 36/25 tests), which is the limit for the Food Quality Control Agency. Similarly, imported polar compound test kits are costly.

To address these limitations and provide a more accessible method for monitoring oil quality, this study aimed to develop an improved test kit for detecting polar compounds in reused frying oils and to compare the formation of polar compounds across different types of cooking oils. A test kit was developed, which was low cost, easy to use and interpret, and did not require special instruments.

## 2. Materials and Methods

This study presents experimental research aimed at developing a test kit for detecting the amount of polar compounds in reused frying oils.

The test kit was developed by modifying the formula of Haskel and Leona [11] to evaluate its efficiency compared to the standard method (GC-column chromatography). The samples were obtained by heating six types of oils: palm oil, soybean oil, canola oil, sunflower oil, rice bran oil, and coconut oil. For each type of oil, 2 L was heated and maintained at a temperature in the range of 170–190 °C. Oil samples were collected every 3 h. Polar compounds were detected using the improved test kit formula developed by the researchers. This process enabled the evaluation of the test kit’s efficiency and the time course of polar compound formation in each oil type.

### 2.1. Test Kit Development

**Figure 1:**
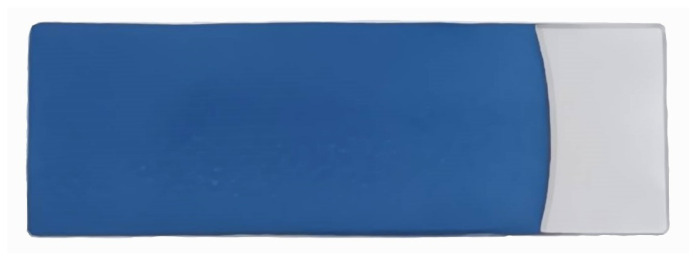
Polar compounds test kit.

The polar compound test kit was developed using the following procedure:

1. In a 250 mL beaker, 100 mL of a methanol (1:1) solution was prepared.
2. A total of 40 g of Silica Gel G Type 60 and 0.1 g of Bromocresol Green were added to the solution.
3. The pH of the solution was adjusted to 7.3 using 0.1 N NaOH, resulting in a blue-colored solution.
4. Clean microscope slides were immersed in the prepared solution and rotated to ensure an even coating.
5. The coated slides were removed and placed horizontally on paper towels to dry for 20 min.
6. The slides were baked in a hot-air oven at 100 °C for 1 h, producing blue-coated slides (Figure 1).
7. The prepared slides were stored in an airtight, opaque container to prevent exposure to air, which could cause the blue coating to turn gray and render the slides unusable.

### 2.2. Evaluation of Test Kit Efficiency Compared to the Standard Method

The fully developed test kit was evaluated under laboratory conditions for its efficiency compared to the standard method. The test kit was used to analyze real samples, including various types of reused frying oils and oils used for frying different raw materials purchased from the food stalls and food shops, totaling 100 samples. The results are compared to the standard method (GC-column chromatography: IUPAC Standard Method 2.507, 1987) [12]. The efficiency of the test kit developed by the researchers was assessed in terms of the accuracy, specificity, sensitivity, positive predictive value, and negative predictive value (Table 1).

The following formulas were used for the calculation:

- Sensitivity = A/(A + C) × 100%.
- Specificity = D/(B + D) × 100%.
- Positive predictive value = A/(A + B) × 100%.
- Negative predictive value = D/(C + D) × 100%.
- Accuracy = (A + D)/(A + B + C + D) × 100%.

## 3. Results

This experimental study utilized the improved test kit to analyze 100 samples of reused frying oils obtained from food stalls and food shops. The results are compared with those obtained using the GC-column chromatography method. The test kit’s efficiency was evaluated, and polar compound levels were categorized. Additionally, the test kit was used to assess six different types of oils subjected to prolonged heating and deep frying. The findings are presented below.

### 3.1. Efficiency of the Polar Compound Test Kit

The performance of the developed test kit for detecting polar compounds in reused frying oils was evaluated against the standard GC-column chromatography method (Table 2).

The developed test kit demonstrated high accuracy and reliability in detecting polar compounds in reused frying oils. The kit can test up to four oil samples simultaneously, with results available within 15 s to 3 min after applying the oil sample to the test strip. The test kit operates based on acid–base reactions. As oil undergoes repeated frying more than five times, its acidity increases. The color change on the test strip indicates the level of polar compounds present:

- Blue: No polar compounds detected (<20% by weight).
- Green/Light green: Polar compounds detected (21–24% by weight).
- Yellow: High levels of polar compounds detected (≥25% by weight).

It is worth noting that highly viscous oils may require longer reading times.

### 3.2. Distribution of Polar Compounds in Six Oil Types

This study examined the time course of polar compound formation in six types of oils: palm oil, soybean oil, canola oil, sunflower oil, rice bran oil, and coconut oil. Each oil type (2000 mL) was heated in the range of 170–190 °C, with samples collected every 3 h for testing. The results for each oil type are detailed below.

#### 3.2.1. Palm Oil

**Figure 2:**
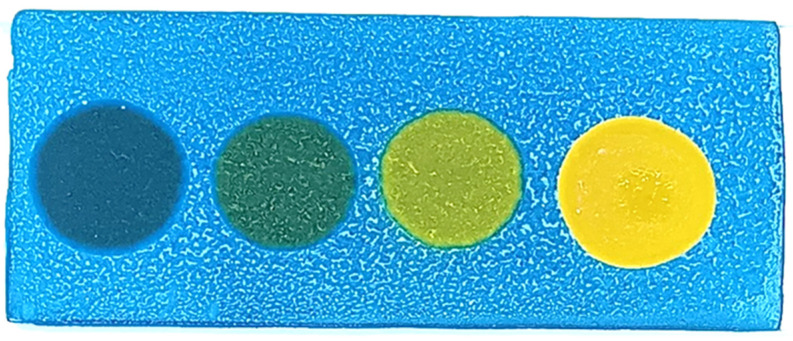
Color changes in the test kit for detecting polar compounds in palm oil.

Palm oil showed the first signs of polar compound formation (20–24%) after 6 h of heating. After 81 h, the polar compound level exceeded 25%, indicating that the oil had degraded and was no longer suitable for use (Table 3 and Figure 2).

#### 3.2.2. Soybean Oil

**Figure 3:**
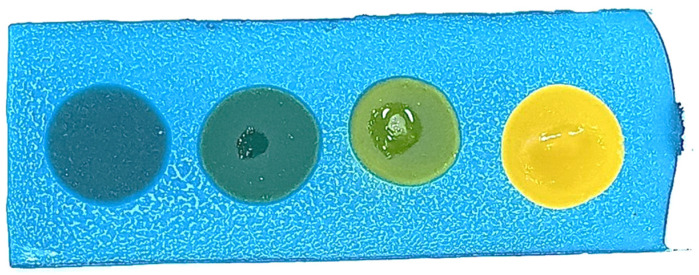
Color changes in the test kit for detecting polar compounds in soybean oil.

Soybean oil maintained low polar compound levels (≤20%) for the first 6 h. Between 9 and 90 h, the levels increased to 21–24%. After 90 h, the polar compound level exceeded 25%, indicating oil degradation (Table 4 and Figure 3).

#### 3.2.3. Canola Oil

**Figure 4:**
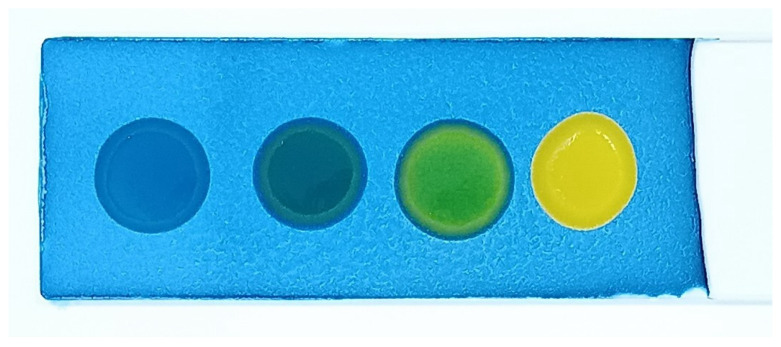
Color changes in the test kit for detecting polar compounds in canola oil.

Canola oil showed low polar compound levels (≤20%) for the first 9 h. Between 10 and 78 h, the levels increased to 21–24%. After 81 h, the polar compound level exceeded 25%, indicating oil degradation (Table 5 and Figure 4).

#### 3.2.4. Sunflower Oil

**Figure 5:**
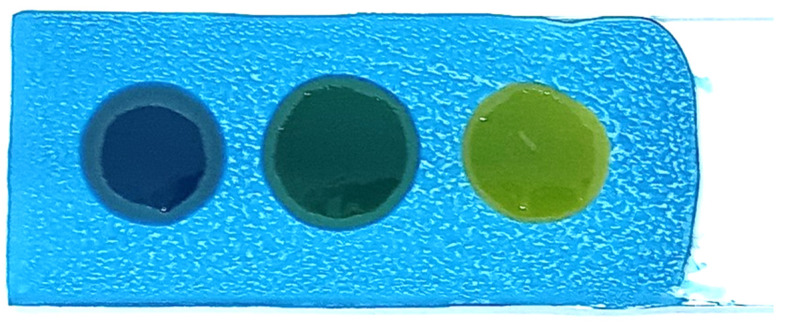
Color changes in the test kit for detecting polar compounds in sunflower oil.

Sunflower oil maintained low polar compound levels (≤20%) for the first 9 h. Between 12 and 90 h, the levels increased to 21–24%. After 90 h, the oil became too viscous for the test kit to detect polar compounds, indicating severe degradation (Table 6 and Figure 5).

#### 3.2.5. Rice Bran Oil

**Figure 6:**
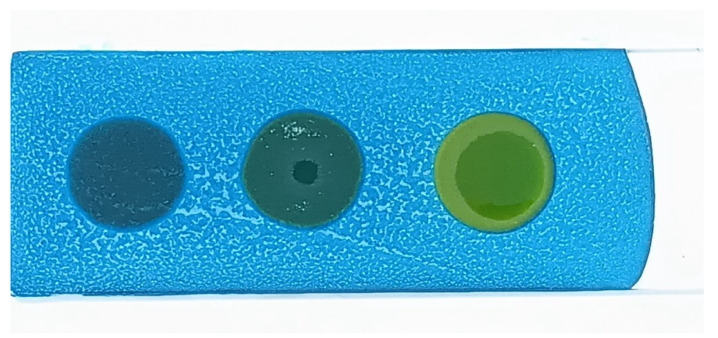
Color changes in the test kit for detecting polar compounds in rice bran oil.

Rice bran oil showed low polar compound levels (≤20%) for the first 6 h. Between 9 and 90 h, the levels increased to 21–24%. After 90 h, the oil became too viscous for the test kit to detect polar compounds, indicating severe degradation (Table 7 and Figure 6).

#### 3.2.6. Coconut Oil

**Figure 7:**
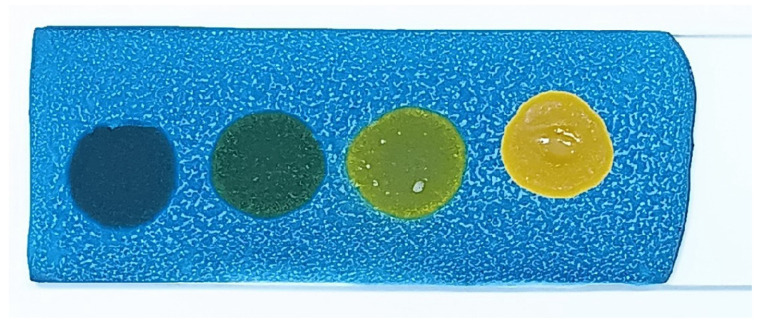
Color changes in the test kit for detecting polar compounds in coconut oil.

Coconut oil maintained low polar compound levels (≤20%) for only the first 3 h. Between 6 and 48 h, the levels increased to 21–24%. After 48 h, the polar compound level exceeded 25%, indicating rapid oil degradation compared to the other oils tested (Table 8 and Figure 7).

## 4. Discussion

The polar compound test kit developed in this study operates on the principle of acid–base reactions. As frying oil is reused over extended periods, its acidity increases. When a sample of used oil is applied to the test slide, the resulting color change indicates the level of polar compounds present. The test kit’s color interpretations are as follows: blue indicates no polar compounds detected (<20% by weight); green indicates polar compounds are in the range of 20–24% by weight; and yellow signifies that polar compounds are ≥25% by weight.

The coloration of frying oil is strongly associated with the concentration of free fatty acids. Yellow color can be an indicator of excessive levels of fatty acid in repeated oil use. Polar compounds are polar degradation products that accumulate in frying oil during the cooking process. When oils are subjected to prolonged heating at high temperatures (approximately 170–190 °C), moisture from food and oxygen from the air accelerate the deterioration of the oil. This degradation occurs primarily through three chemical reactions: hydrolysis, oxidation, and polymerization. These reactions lead to the formation of both volatile and non-volatile compounds in large quantities, which significantly alter the physical properties of the oil—such as changes in color, odor, and flavor; a decrease in the smoke point; and an increase in viscosity.

One limitation of the test kit is that highly viscous oils may require a longer time to yield accurate results. Oils with polar compound levels exceeding 25% by weight are known to contain high concentrations of carcinogenic substances and pose significant health risks to consumers. This is why the Ministry of Public Health in Thailand has set the maximum allowable limit for polar compounds in frying oils at 25% by weight [9].

The study of polar compound formation in six different types of frying oils (palm oil, soybean oil, rice bran oil, sunflower oil, canola oil, and coconut oil) revealed varying time courses for polar compound accumulation. Palm oil showed the first signs of polar compound formation (21–24%) after 6 h of heating, while soybean oil and rice bran oil maintained lower levels for up to 90 h [13]. The total polar compound content in frying oil depends on factors such as frying time, temperature, the type of food being fried, the frying load, and the type of oil used [14,15].

When subjected to prolonged heating under laboratory conditions, all six oil types underwent significant changes. The oils experienced important reactions, including oxidation, hydrolysis, polymerization, and pyrolysis [16]. These reactions resulted in changes in color, increased viscosity, rancid odor development, foaming, and reduced smoke points, all of which are indicative of oil degradation [17,18].

The varying rates of polar compound formation among different oil types can be attributed to their differing fatty acid compositions, particularly the ratio of saturated-to-unsaturated fatty acids. Oils with higher levels of unsaturated fatty acids are generally less stable and more prone to oxidation and degradation when exposed to prolonged heat [14,15]. Palm oil consists mainly of palmitic acid and oleic acid, offering a balance between thermal stability and oxidative susceptibility. Soybean oil, by contrast, contains high levels of polyunsaturated fatty acids, particularly linoleic acid and alpha-linolenic acid, which make it more prone to oxidative degradation. The polar compound levels in soybean oil tend to increase oil degradation compared to larger fryers. Fatty acid analysis was used to determine effects on hydrolysis in the frying oils [4].

Coconut oil is highly saturated, with lauric acid as the predominant fatty acid. The thermal instability of these fatty acids under prolonged heating conditions leads to the rapid breakdown and accumulation of polar compounds [1]. Canola oil is rich in oleic acid and contains low saturated fats, contributing to its thermal stability. The moderate levels of polyunsaturated fatty acids also allow canola oil to resist rapid degradation, making it suitable for repeated frying, as confirmed by its slower accumulation of polar compounds. Sunflower oil is high in linoleic acid, making it less stable under high heat unless it is a high-oleic variety. Rice bran oil offers a balanced profile, with both oleic and linoleic acids in moderate proportions [19]. This composition likely contributed to the delayed accumulation of polar compounds. Fatty acid content increased with increasing frying time in all oils.

Based on the findings of this study, coconut oil appears to be the least suitable for deep frying, as it rapidly accumulates polar compounds. Conversely, rice bran oil, sunflower oil, canola oil, and palm oil were found to be more suitable for frying, with significant polar compound contamination occurring only after more than 80 h of use. However, it is crucial to note that food service operators should avoid mixing fresh oil with used oil to extend its usability, as this practice can compromise food safety and quality.

The developed test kit provides a quick and accessible method for monitoring oil quality, enabling more frequent and convenient testing compared to traditional laboratory methods. This allows food service operators to make informed decisions about when to replace their frying oils, balancing cost considerations with food safety and quality. While the polar compound content is a critical indicator of oil quality, it is important to consider other factors as well. These include the oil’s color, viscosity, odor, and smoke point. Food service operators should be vigilant in monitoring oil quality, especially after five-or-more uses, as various factors can accelerate polar compound formation [14].

## 5. Conclusions

The results of detecting polar compounds in reused frying oils across six oil types (palm oil, soybean oil, canola oil, sunflower oil, rice bran oil, and coconut oil), controlled at a temperature in the range of 170–190 °C, showed that the time required to reach critical polar compound levels varied between oil types. These differences arise due to variations in the composition of saturated and unsaturated fatty acids in each oil. Oils with a higher unsaturated-fatty-acid content are less stable and more prone to degradation when exposed to prolonged heat, leading to increased viscosity.

Palm oil showed yellow results after 81 h of heating, indicating polar compound levels of ≥25% by weight. Soybean oil demonstrated greater stability, showing yellow results only after more than 90 h of heating, indicating that polar compound levels had exceeded 25% by weight. Similarly, canola oil showed yellow results after 81 h of heating, indicating polar compound levels of ≥25% by weight. Sunflower oil maintained polar compound levels within the range of 10–24% by weight up to 90 h, but beyond this point, the oil became too viscous for the test kit to provide interpretable results. For rice bran oil, light-green results were observed at 99 h of heating, indicating polar compound levels in the range of 21–24% by weight; however, beyond 90 h, the oil’s viscosity increased to the extent that the test kit could no longer detect polar compounds. Coconut oil was found to degrade the most rapidly, showing yellow results after only 48 h of heating, which indicated polar compound levels of ≥25% by weight.

The improved polar compound test kit demonstrated the ability to effectively detect polar compounds in reused frying oils. The variation in oil degradation rates highlights the importance of considering oil type when monitoring frying-oil quality. Coconut oil was found to degrade the most rapidly, whereas rice bran oil, sunflower oil, canola oil, and soybean oil exhibited greater stability. Future improvements to the test kit could enhance its accuracy and efficiency, making it an even more valuable tool for monitoring oil quality in both laboratory and field settings.

## Figures and Tables

**Table 1:** The efficiency of the polar test kit.

Test Kit	Reference Method (GC)	
	Positive	Negative
Positive (≥ 25%)	True positive (A)	False positive (B)
Negative (< 25%)	False negative (C)	True negative (D)
Total	N^+^ = (A + C)	N^−^ = (B + D)

**Table 2:** Efficiency of the polar compound test kit for reused frying oils (100 samples).

**Test kit**	**GC-column chromatography**		Accuracy = 92.00% Sensitivity = 88.09% Specificity = 94.82% Positive predictive value = 92.50% Negative predictive value = 91.66%
	**+**	**−**	
+(≥25%) *	37	3	
−(<25%) **	5	55	

* ≥25% polar compounds by weight (positive), ** <25% polar compounds by weight (negative).

**Table 3:** Time course of polar compound formation in palm oil.

Time (h)	Test Kit Color	Polar Compounds (%)	Interpretation
0–3	Blue	≤20	Safe to use
6–78	Green/Light green	21–24	Approaching degradation
>81	Yellow	≥25	**“Do not use”**

**Table 4:** Time course of polar compound formation in soybean oil.

Time (h)	Test Kit Color	Polar Compounds (%)	Interpretation
0–6	Blue	≤20	Safe to use
9–90	Green/Light green	21–24	Approaching degradation
>90	Yellow	≥25	**“Do not use”**

**Table 5:** Time course of polar compound formation in canola oil.

Time (h)	Test Kit Color	Polar Compounds (%)	Interpretation
0–9	Blue	≤20	Safe to use
10–78	Green/Light green	21–24	Approaching degradation
>81	Yellow	≥25	**“Do not use”**

**Table 6:** Time course of polar compound formation in sunflower oil.

Time (h)	Test Kit Color	Polar Compounds (%)	Interpretation
0–9	Blue	<20	Safe to use
12–90	Green/Light green	20–24	Approaching degradation
>90	Not detectable	Not Detectable *	**“Do not use”**

* Not detectable due to high viscosity.

**Table 7:** Time course of polar compound formation in rice bran oil.

Time (h)	Test Kit Color	Polar Compounds (%)	Interpretation
0–6	Blue	≤20	Safe to use
7–90	Green/Light green	21–24	Approaching degradation
>90	Not detectable	Not Detectable	**“Do not use”**

**Table 8:** Time course of polar compound formation in coconut oil.

Time (h)	Test Kit Color	Polar Compounds (%)	Interpretation
0–3	Blue	≤20	Safe to use
4–48	Green/Light green	21–24	Approaching degradation
>48	Yellow	≥25	**“Do not use”**

## Data Availability

The original contributions presented in this study are included in the article/Supplementary Material. Further inquiries can be directed to the corresponding author.

## Supplementary Materials

The following supporting information can be downloaded at: https://www.mdpi.com/article/10.3390/foods14091572/s1, Table S1: Interpretation of polar compound using the standard method (GC-column chromatography) and the polar compounds test kit.

## Abbreviations

The following abbreviations are used in this manuscript:

mL	Milliliter
GC	Gas Column Chromatography
NaOH	Sodium Hydroxide
IUPAC	International Union of Pure and Applied Chemistry
