# Supplementary material for: Modified Test Kit for Detecting Polar Compounds and Evaluating Their Distribution in Reused Frying Oil
Source: Foods. 2025 Apr 29;14(9):1572. doi: 10.3390/foods14091572 (PMC12071793; doi:10.3390/foods14091572)
Supplement: Supplementary file 1 [file foods-14-01572-s001.zip › foods-3521398-supplementary.pdf]

**Table S1.** Interpretation of polar compound using the standard method (GC-column chromatography) and the polar compounds test kit.

| No. Sample | Polar value GC (%) | Result (≥25/+, <25/-) | | Color | No. Sample | Polar value GC (%) | Result (≥25/+, <25/-) | | Color |
|---|---|---|---|---|---|---|---|---|---|
| | | GC | Test kit | | | | GC | Test kit | |
| 1 | 7.4 | - | - | Blue | 51 | 35.5 | + | + | Yellow |
| 2 | 5.1 | - | - | Blue | 52 | 16.8 | - | - | Blue |
| 3 | 6.3 | - | - | Blue | 53 | 4.7 | - | - | Blue |
| 4 | 13.2 | - | - | Blue | 54 | 5.4 | - | - | Blue |
| 5 | 27.3 | + | - | Green | 55 | 8.5 | - | - | Blue |
| 6 | 6.5 | - | - | Blue | 56 | 14.5 | - | - | Blue |
| 7 | 4.1 | - | - | Blue | 57 | 5.2 | - | - | Blue |
| 8 | 12.7 | - | - | Blue | 58 | 6.9 | - | - | Blue |
| 9 | 6.4 | - | - | Blue | 59 | 9.2 | - | - | Blue |
| 10 | 4.1 | - | - | Blue | 60 | 14.7 | - | - | Blue |
| 11 | 2.2 | - | - | Blue | 61 | 9.8 | - | - | Blue |
| 12 | 19.9 | - | - | Blue | 62 | 8.8 | - | - | Blue |
| 13 | 9.4 | - | - | Blue | 63 | 10.7 | - | - | Blue |
| 14 | 27.6 | + | - | Light green | 64 | 9.9 | - | - | Blue |
| 15 | 25.9 | + | - | Light green | 65 | 16.4 | - | - | Blue |
| 16 | 24.4 | + | + | Light green | 66 | 8.8 | - | - | Blue |
| 17 | 20.2 | + | + | Green | 67 | 7.7 | - | - | Blue |
| 18 | 20.6 | + | + | Green | 68 | 10.5 | - | - | Blue |
| 19 | 20.8 | + | + | Green | 69 | 10.1 | - | - | Blue |
| 20 | 20.5 | + | + | Green | 70 | 10.1 | - | - | Blue |
| 21 | 20.3 | + | + | Green | 71 | 15.5 | - | - | Blue |
| 22 | 20.8 | + | + | Green | 72 | 23.9 | + | + | Green |
| 23 | 20.7 | + | + | Green | 73 | 15.6 | - | - | Blue |
| 24 | 22.3 | + | + | Green | 74 | 11.8 | - | - | Blue |
| 25 | 22.1 | + | + | Green | 75 | 4.7 | - | - | Blue |
| 26 | 21.7 | + | + | Green | 76 | 5.2 | - | - | Blue |
| 27 | 21.4 | + | + | Green | 77 | 11.6 | - | - | Blue |
| 28 | 21.3 | + | + | Green | 78 | 6.5 | - | - | Blue |
| 29 | 20.7 | + | + | Green | 79 | 9.4 | - | - | Blue |
| 30 | 20.6 | + | + | Green | 80 | 14.5 | - | - | Blue |
| 31 | 20.2 | + | + | Green | 81 | 9.2 | - | - | Blue |
| 32 | 20.6 | + | + | Green | 82 | 5.4 | - | - | Blue |
| 33 | 21.1 | + | + | Green | 83 | 6.8 | - | - | Blue |
| 34 | 21 | + | + | Green | 84 | 13.5 | - | - | Blue |
| 35 | 21.1 | + | + | Green | 85 | 9.8 | - | - | Blue |
| 36 | 21.4 | + | + | Green | 86 | 8.4 | - | - | Blue |
| 37 | 30.4 | + | + | Yellow | 87 | 10.4 | - | - | Blue |
| 38 | 25.6 | + | + | Yellow | 88 | 9.8 | - | - | Blue |
| 39 | 50.7 | + | + | Yellow | 89 | 30.5 | + | - | Light green |
| 40 | 51.1 | + | + | Yellow | 90 | 7.7 | - | - | Blue |
| 41 | 52.6 | + | + | Yellow | 91 | 27.5 | + | - | Light green |
| 42 | 61.7 | + | + | Yellow | 92 | 4.8 | - | - | Blue |
| 43 | 70.4 | + | + | Yellow | 93 | 10.8 | - | - | Blue |

| No. Sample | Polar value GC (%) | Result (≥25/+, <25/-) | | Color | No. Sample | Polar value GC (%) | Result (≥25/+, <25/-) | | Color |
|---|---|---|---|---|---|---|---|---|---|
| | | GC | Test kit | | | | GC | Test kit | |
| 44 | 60.2 | + | + | Yellow | 94 | 15.5 | - | + | Yellow |
| 45 | 47.4 | + | + | Yellow | 95 | 15.6 | - | - | Blue |
| 46 | 23.3 | + | + | Green | 96 | 11.8 | - | + | Green |
| 47 | 25.3 | + | + | Yellow | 97 | 16.4 | - | - | Blue |
| 48 | 35.8 | + | + | Yellow | 98 | 4.7 | - | - | Blue |
| 49 | 25.5 | + | + | Yellow | 99 | 3.5 | - | - | Blue |
| 50 | 24 | + | + | Light green | 100 | 13.2 | - | + | Green |
